# Early Trajectory Prediction in Elite Athletes

**DOI:** 10.1007/s12311-018-0975-9

**Published:** 2018-09-14

**Authors:** Cullen B. Owens, Casper de Boer, Giulia Gennari, Robin Broersen, Johan J. Pel, Brian Miller, Wesley Clapp, Ysbrand D. van der Werf, Chris I. De Zeeuw

**Affiliations:** 1 Department of Neuroscience, Erasmus MC, 3000 DR Rotterdam, The Netherlands; 2 NeuroScouting LLC, 234 Broadway Unit 2, Cambridge, MA 02139 USA; 3 Institute for Molecular and Cell Biology, Agency for Science, Technology, and Research (ASTAR), 61 Biopolis Dr, Singapore, 138673 Singapore; 4 University of Padua, via Venezia 12, 35131 Padua, Italy; 5 Netherlands Institute for Neuroscience, Royal Academy of Arts & Sciences, 1105 BA Amsterdam, The Netherlands; 6 Amsterdam UMC, Vrije Universiteit Amsterdam, Department of Anatomy & Neurosciences, Amsterdam Neuroscience, de Boelelaan, 1117 Amsterdam, Netherlands

**Keywords:** Trajectory prediction, Decision-making, Psychophysics, Elite athletes, Baseball, Cerebellum, fMRI, Pupillary response, Optimal feedback control

## Abstract

Cerebellar plasticity is a critical mechanism for optimal feedback control. While Purkinje cell activity of the oculomotor vermis predicts eye movement speed and direction, more lateral areas of the cerebellum may play a role in more complex tasks, including decision-making. It is still under question how this motor-cognitive functional dichotomy between medial and lateral areas of the cerebellum plays a role in optimal feedback control. Here we show that elite athletes subjected to a trajectory prediction, go/no-go task manifest superior subsecond trajectory prediction accompanied by optimal eye movements and changes in cognitive load dynamics. Moreover, while interacting with the cerebral cortex, both the medial and lateral cerebellar networks are prominently activated during the fast feedback stage of the task, regardless of whether or not a motor response was required for the correct response. Our results show that cortico-cerebellar interactions are widespread during dynamic feedback and that experience can result in superior task-specific decision skills.

## Introduction

The cerebellum is an important site of plasticity for motor learning and part of a larger network consisting of both cortical and subcortical brain areas that support functions such as adaptation of movements, temporal processing [1], and spatiotemporal prediction [2]. Increasing evidence has surfaced indicating that the cerebellum may also moderate cognitive control in both humans and animals when strict temporal processing is required [3–5].

It has been said that hitting a major league fastball is one of the most biologically challenging tasks for a human to accomplish. It demands years of practice to execute at the highest level and requires millisecond level precision in neurocomputational terms. In less than 500 ms, the batter must watch the ball coming out of the pitcher’s hand (preparatory period), recognize the pattern of the seams on the ball and interpolate the spin (pattern recognition), and then integrate that information with the expected trajectory and speed of the pitch (timed trajectory prediction) in order to determine whether it will pass through the strike zone or not (a go/no-go decision). If the ball appears to pass through the strike zone, the batter must make fast and accurate motor adjustments with bat in hand to make proper contact with the ball (online motor control). Therefore, the act of hitting in baseball consists of discrete trials that require several categorically different sequential cognitive and motor processes in a very short amount of time.

Specifically, hitting a baseball can be divided into two main subtasks of a different nature. The first 225 ms is considered a go/no-go timed decision-making task, since the batter has to decide in this time window whether or not to swing. The latter part of the pitch trajectory is considered a motor control and timing task, because the batter has to make fast motor adjustments to contact the ball accurately [6]. Given this clear segregation, baseball is an attractive model for dissecting the differential impact of various temporal cues on perception, decision-making, and sensorimotor control.

There is ample scientific evidence that elite athletes develop task specific skills in perception, cognition, and motor control [7, 8]. For example, well-practiced baseball players show improved reaction times in a go/no-go task with stationary cues. They also show an increased ability to extrapolate the momentum of a visually occluded moving target [9, 10]. Furthermore, expert cricket batsmen making predictive saccades during the pitch show that they make better use of early flight information than novices [11]. However, in these examples, the relationship between psychophysical and neurophysiological demands is less well understood. Although it has been posited that elite athletes may bias the correct action earlier and that elite athlete action selection is superior, it has to our knowledge not been shown how psychophysical and neurophysiological computations may facilitate superior performance in sequence, i.e., from preparatory period, to trajectory prediction to decision making and finally to action selection. We chose to investigate the parametrical space of this sequence and the functional neural network that might mediate it.

## Materials and Methods

### Participants

In total, 10 male baseball field players (mean age 17.4 years) defined as having seen > 5000 live game pitches from the AAA national youth team of the Royal Netherlands Baseball and Softball Federation (KNBSB) and 10 male age-matched non-baseball players (mean age 18.3 years); [*t* (18) = 0.952, *P* = .354]) were included. Experts and controls were excluded from the study if they reported playing video games more than 5 h per week, had any known motor problems, or did not have normal or corrected-to-normal vision. In the latter case, subjects’ vision was tested using a standard eye chart. Prior to measurements, a written informed consent was obtained from all subjects. In the case of subjects under the age of 18 years, signed informed consent was obtained from both parents. The study was approved by the medical ethical committee of Erasmus MC, Rotterdam (MEC-2012-524). One control and one expert subject were excluded from pupillary response analysis, and one expert subject was excluded from initiation of hand movement analysis due to insufficient quality of data.

### Study Design

All subjects performed a simple reaction time task (RTT) and a trajectory prediction task (TPT), divided across four blocks in a fixed order. Subjects first performed 50 trials of the RTT on measurement setup 1, and then 3 blocks of 80 trials of TPT on measurement setup 2. Both setups were situated in a temperature controlled dark room and all subjects were given a few minutes of rest between blocks.

#### RTT

Measurement setup 1 was used to calculate the reaction time. The RTT consisted of a 25-in. monitor (IIyama, Nagano, Japan, 60 Hz) and a keyboard. During RTT subjects placed their head on a chin-rest in front of the screen at a distance of 460 mm, which resulted in a visual angle of 59° × 36° (width × height). The subjects saw a video demo of the task prior to the measurements, and they were instructed to press the space bar of a keyboard as quickly as possible when the visual stimulus (a white ball with red seams on a black background) appeared. Each subject received a total of 50 trials with a short break after 25 trials. During each trial, the stimulus was displayed for 500 ms with random intertrial intervals between 1.5 and 4 s. The maximum allowed reaction time was 750 ms, and key presses in the first 125 ms were considered catch trials. For each RTT trial, reaction time was calculated as the time between stimulus presentation and pressing the keyboard (excluding the catch time trials). After each trial, the subject’s reaction time was presented above the stimulus to motivate the subject to decrease their reaction time.

#### TPT

Measurement setup 2 consisted of a combination of a 32-in. touch screen (ELO Touchsystems, Leuven, Belgium), a 3D infrared motion capture system (Vicon Model, Oxford, UK), and an infrared eye tracking system (Chronos Vision, Berlin, Germany). Eye and hand movements were recorded at a rate of 200 Hz. The exact specifications of this setup have been published (de Boer et al. 2013). Subjects placed their head on a chin-rest in front of the screen at a distance of 460 mm, resulting in a visual angle of 75° × 46° (width × height). Prior to the measurements, all subjects received verbal instructions followed by a series of practice runs, with a maximum of 20 trials.

**Fig. 1:**
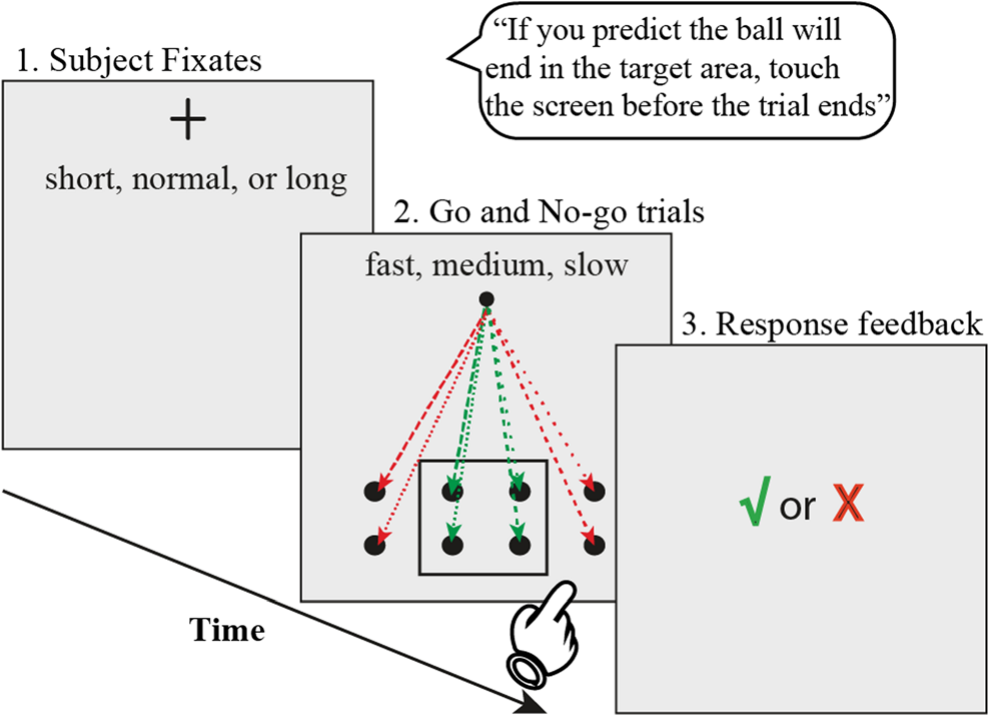
Trajectory prediction task. A fixation cross appeared with a variable duration (390 ms, 890 ms, or 1900 ms), followed by either a fast (385 ms), medium (485 ms), or slow (585 ms) moving stimulus to one of eight final locations, within (green dotted lines; go trials) or outside the target square (red dotted lines; no-go trials). Fixation duration and stimulus speed and trajectory were pseudo-randomly intermixed. A screen touch (and hold) in go trials (hit) and no-touch in no-go trials (correct rejection) resulted in a correct response (green check). A red X appeared after an incorrect response: screen touch during no-go trials (false alarm) or no screen touch during go trials (miss). Each subject performed three blocks of 80 trials on the behavioral task

In this task (Fig. 1), subjects were instructed to fixate on a black cross, located at a 20° vertical visual angle above the center of the screen. After a variable fixation period, the cross was replaced by a solid black circle that immediately started moving from the location of the cross toward a target area (10° × 10°) at the bottom of the screen in a straight-line trajectory. If subjects anticipated the stimulus would end up inside the target area (go trial), they were instructed to touch the screen as quickly as possible with their dominant index finger and hold it there. If subjects anticipated the stimulus would end up outside the target area (no-go trial), subjects were instructed not to touch the screen. It was emphasized that subjects had to touch and hold the position on the screen before the stimulus reached its final position. After the stimulus had reached its final position, visual feedback on trial performance (correct or incorrect) was presented for 500 ms.

The following parameters were pseudo-randomly varied and counterbalanced within each block. Four *final locations* were designated as “go” trials, and four locations were designated as “no-go” trials (final locations outside the target area on both sides). Three stimulus *flight speeds* were designated (385 ± 5, 485 ± 5, or 585 ±5 ms) and were presented 24 times per speed (three times per final location). A stimulus flight time of 985 ms (probe) was used eight times in total (one time per final location). *Presentation time of the fixation cross* was either 390 ± 5, 890 ± 5, or 1900 ± 5 ms. Each fixation duration was applied 24 times per block. During stimulus flight speeds of 985 ms (eight trials), a fixation duration of 1000 ms was applied. Neighboring final locations were equidistant vertically and horizontally to each other, forming a 4 × 2 grid. All final locations were targeted 10 times per block. All stimuli varied ± 5 ms in duration due to an asynchrony between the stimulus presentation rate (200 Hz, synchronized with the eye-tracking system and the motion detection system) and the screen refresh rate (60 Hz). For brevity, we refer to each flight speed as 390 ms (fast), 490 ms (medium), and 590 ms (slow), and we refer to each presentation time of the fixation cross as 390 ms (short), 890 ms (normal), and 1900 ms (long).

### Data Processing and Outcome Parameters

At least 10 reliable trials were required in order for an outcome parameter to be included in final analysis. Seven eye movement outcomes, two hand outcomes, and eight pupil outcomes were calculated. Otherwise, the subject was excluded from the analysis.

*Total tracking time* is the total time per trial (in ms and as percentage of the total trial time) during which gaze was < 8° from the stimulus. *Mean gaze to stimulus distance* (GSD) is the mean distance (in degrees) between the subject’s gaze and position of the stimulus during the part of the trial that is defined as “tracking.” *Eye response time* is defined as the time between stimulus onset and first saccade onset (gaze velocity exceeding 50°/s). The *primary saccade amplitude* is defined as the degrees of the first saccadic eye movement. The *fixation error* is the mean number of degrees from the fixation cross across the entire fixation period. *Hand response time* is the time between stimulus onset and index finger velocity exceeding 20°/s. *Touch time* is the time between stimulus onset and touching the screen. *Trial performance* calculated as percentage of correct or incorrect trials is classified as a hit (correct go trials), miss (incorrect go trials), correct rejection (correct no-go trials), or false alarm (incorrect no-go trials). For pupillary responses, we calculated mean pupil *diameter* (in millimeters) at *baseline* (first 100 ms of the trial), *end of the fixation* period (last 100 ms), and *end of the stimulus* presentation (last 100 ms). Additionally, we computed the slope (i.e., steepness) of diameter change across the entire trial (*slope overall*), during the *fixation* period only, and during the stimulus *flight* only. Finally, the amplitude of the pupillary response is indexed by the difference between maximal pupil diameter within the whole trial and baseline diameter (*peak size change*) and the time at which such maximal diameter was reached (*latency to peak*). Eye and hand traces were visualized using custom MATLAB (MathWorks, Natick, MA, USA) software. Using these visualizations, each trial was manually checked while the rater was blind to subject and group. In each trial, *x*- and *y*-coordinates of the eye movements, gaze and hand velocity, and task-evoked pupillary response were plotted in relation to the stimulus onset and subject’s screen touch. All trials that did not allow reliable analysis (e.g., due to blinking or noisy signal) were removed.

### Statistical Analysis

To assess for normality of distributions, we used the Shapiro-Wilk test. When normality was met, we tested for differences in all outcome parameters between experts and controls using univariate ANOVA tests with group as between-subject variable and block, final location, fixation duration, and speed as within-subject variable. To test for a within-subject effect of block on each parameter, repeated measures ANOVA tests were used. Post-hoc Bonferroni-corrected and independent samples *t* tests were used to more closely examine differences between groups and within individual blocks. For comparisons where data were not normally distributed, we used the Mann-Whitney *U* test. In all statistical tests, significance level was set at 0.05. All statistical testing was performed in IBM SPSS Statistics 21. *T* tests are two-way unless stated otherwise. Results are calculated as mean ± standard deviation (SD), unless stated otherwise and figures display standard error of the mean (SEM)*.*

## fMRI Methods

### Procedure

We used an adapted version of our novel timed, trajectory prediction task for fMRI investigation. For the MRI implementation of the task, we restricted the presentation of the stimuli to two speeds only, i.e., fast (370 ± 20 ms) and medium (500 ± 10 ms), to maintain sufficient power for event-related analysis. We also increased the number of trials from 80 trials per block to 96 trials per block. All other stimuli parameters were the same as that described in the behavioral study. We recruited six male right-handed participants (two expert baseball players and four novices) from the same cohort as in the behavioral study.

Subjects were lying in the MRI scanner in a supine position. The visual stimuli were provided by using an LCD projection onto a screen standing behind the MR scanner; the image of this projection was visible through a mirror attached to the head coil above the subjects’ eyes. Responses of the right hand were acquired using a four-button MR-compatible response box (Current Design, HHSC-1x4-CL). Every subject performed three blocks of the task, with 48 go and 48 no-go trials each.

### Imaging Parameters and Acquisition

Functional and structural MR scans were recorded using a 3T scanner (Philips, 3.0-Tesla Achieva) at the Spinoza Center, University of Amsterdam. For functional MRI scans, whole-brain functional T2*-weighted MRI data were acquired using a gradient-echo planar imaging (EPI) sequence (55 transverse slices with 0 mm gap, ascending slice acquisition; voxel size 2.5 × 2.5 × 2.5 mm^3^; repetition time (TR) 3179 ms, echo time (TE) 29.93 ms; flip angle 80°; field of view (FOV) 200 × 200 mm^2^). For co-registration purposes, we acquired T1-weighted MR images (220 slices; TR 8.2 ms; TE 3.8 ms; inversion time 670.4 ms; FOV 240 × 188 mm^2^, matrix size 240 × 187; flip angle 8°; voxel size 1 × 1 × 1 mm^3^). Head movements were minimized by restraining the subject’s head using sponge cushions inside the 32-channel head-coil.

### Analysis

All pre-processing and analysis steps were done using FSL 5.08 (FMRIB’s Software Library, www.fmrib.ox.ac.uk/fsl). In brief, we performed skull-stripping of the T1 images using BET, filtered the functional images using a 100-s high-pass filter and smoothed them with a 5-mm full-width at half maximum (FWHM) Gaussian kernel. We performed event-related general linear modeling (GLM) at first level on the three blocks per subject, using FEAT (FMRI Expert Analysis Tool) version 6.00, part of FSL. Our first analysis investigated brain activation patterns associated with the different response types, i.e., go and no-go responses. In the model, we used hits, correct rejections, misses, and false alarms as events. We modeled the events for the duration of the stimulus. Using these events, we constructed the following contrasts: “correct hits–misses” (go contrast) and “correct rejections–misses” (no-go contrast). We generated first-level GLM analyses contrasting the responses to fast vs. medium stimuli, across the combination of correct hits and correct rejections, by creating a “fast–medium” contrast. In addition, we generated separate fast–medium contrasts for the correct hits and correct rejection stimuli, respectively.

Activation maps were co-registered to the individual T1 scan using BBS and then brought into standard space using linear warping with 12 degrees of freedom.

We then carried each of the three different types of first-level analyses into second level using a fixed-effects higher-level analysis for the three runs per subject, thereby constructing single-subject activation maps. These images were then carried into third-level mixed-effects analyses using FLAME1 + 2 to be maximally sensitive to meaningful effects in small numbers of subjects, so as to obtain group averages via a one-sample *t* test. The main effects of activation are reported at a *z* threshold of 2.3 with cluster correction at *P* < 0.01 (Worsley 2001). All images conform to neurological convention, i.e., the left side is left in the image.

## Results

### Experts Perform Better Only Under the Most Challenging Conditions

**Fig. 2:**
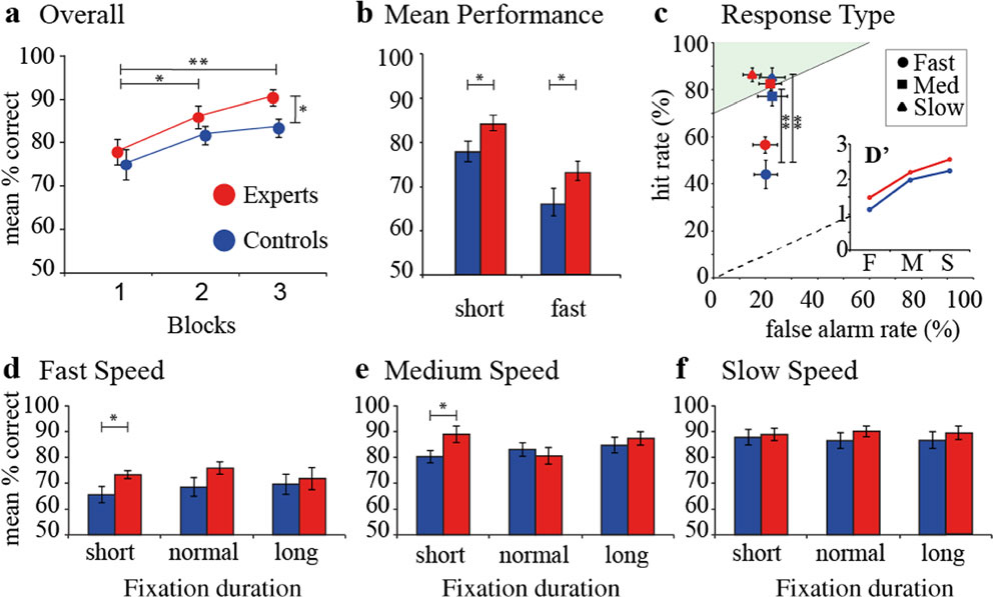
Performance. **a** Overall group average performance over 3 blocks of 80 trials. Subjects improved from block 1 to block 2 and block 1 to block 3. Experts performed significantly better than controls on block 3. **b** Experts perform better on average during the shortest fixation duration and at the fastest flight times. **c** Experts do better than controls as flight speed decreases by increasing hit rate with a higher *D*′ score (inset, F fast, M medium, S slow). **d–f** When all blocks are compiled and broken down by fixation duration and flight time, experts perform better at the short fixation duration of the fast (**d**) and medium (**e**) flight times, but not the slow. (*P < .05; **P < .01) (*N* = 10/group). Numbers represent means and SEM

We first investigated subjects’ overall performance, i.e., percentage of correct trials, over all three blocks. Both experts and controls improved their overall performance over blocks of trials [*F* (1, 18) = 21.042, *P* < .001]). *Post-hoc* Bonferroni corrected tests showed that performance in block 2 (mean difference (MD) = 7.2, *P* = .033) and block 3 (MD = − 10.2, *P* = .001) was better than performance in block 1. We found no interaction effect between block and group on overall performance, indicating that both experts and controls performed better in blocks 2 and 3 compared to block 1. However, experts performed better than controls in block 3 (MD = − 6.70, *t* (18) = − 2.435, *P* = .026), but not in block 1 or 2 (Fig. 2a).

At the most challenging temporal constraints, i.e., when fixation time prior to the trial was short, experts performed on average 6.2% better (*t* (18) = 2.154, *P* = .023). In trials where stimulus speed was fast, experts performed on average 7.2% better (*t* (18) = 1.917, *P* = .035) (Fig. 2b).

We also tested what type of responses contributed to task performance relevant to flight speed and group. Subjects had a better performance at slow and medium flight speeds compared to fast flight speeds due to increased hit responses [*F* (2, 54) = 44.157, *P* < .001] rather than reduced false alarms [*F* (2, 54) = 0.592, *P* = .557] (Fig. 2c). *Post-hoc* Bonferroni-corrected tests showed that hit rate was lower in trials with fast stimulus speeds compared to trials with medium stimulus speeds [MD = − 0.294, *P* < .001] and slow stimulus speeds [MD = − 0.346, *P* < .001]. Differences between groups were also reflected in the *D*′ measure, a statistical measure in signal detection theory to quantify how a system distinguishes signal from noise. We found experts with higher *D*′ scores overall [controls 1.14, 1.99, 2.25; experts 1.48, 2.19, 2.57], and both groups show reductions in hits rather than increased false alarms when the flight speed increases, highlighting the impact of flight speed on subjects’ stimulus sensitivity rather than specificity (Fig. 2c, inset).

Finally, we were interested to see how groups differed in all nine trial variations. Here we compiled performance over all three blocks, measuring performance during all combinations of fixation duration *and* stimulus flight speeds. When fixation duration were short, experts performed better during fast [controls 65.4 ± 3.1%, experts 73.1 ± 1.6%, *t* (18) = − 2.216, *P* = .040] (Fig. 2d) and medium (Fig. 2e) flight speeds [controls 80.2 ± 2.5%, experts 88.9 ± 3.2%, *t* (18) = − 2.145, *P* = .046]. These differences did not hold for other combinations (Fig. 2d–f).

### Fast Decision-Making Was Associated with Activation of Cerebellar Areas

**Fig. 3:**
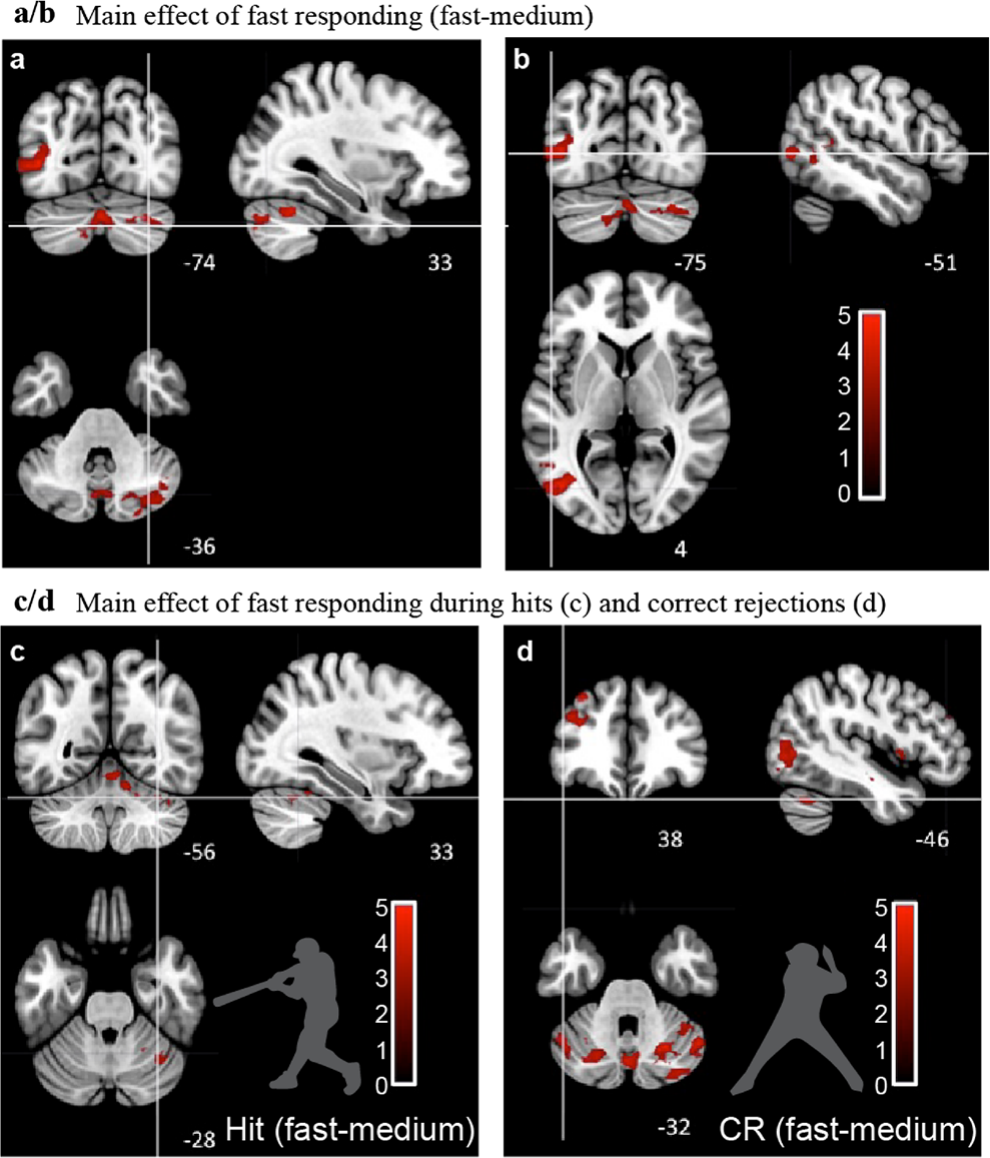
fMRI. The main effect of fast responding is shown in **a** and **b**. Fast responding is associated with medial and lateral cerebellum. When contrasting BOLD response during fast vs. medium stimuli, across combined hits and correct rejections, differential activity was associated with medial cerebellum (vermis), right lateral cerebellum (crus areas), and left inferior parietal lobe (and Table 1). **c** BOLD response differences of hit trials between medium and fast flight speed. Cerebellar vermis and right cerebellar cortex (area I–VII, crus I) showed a large significant cluster (*Zmax* = 3.81; *x* = 6, *y* = − 54, *z* = − 12). Images were thresholded at *z* > 2.3 cluster corrected at *P* = .01; only the vermis and right cerebellum exceeded the significance threshold. **d** BOLD response differences of correct rejections between fast and medium flight speed in six large clusters including cerebellar vermis, right cerebellar cortex (areas I–VII, crus I) (*Zmax* = 4.19); left putamen, left frontal operculum (*Zmax* = 4.1); left and right temporo-occipital cortex, inferior parietal lobule (*Zmax* = 4.22 and 3.96); bilateral superior parietal gyrus, intraparietal sulcus (*Zmax* = 4.07); and middle frontal gyrus (*Zmax* = 4.25) (*N* = 6)

Using fMRI, we investigated which brain areas were engaged during our task. We hypothesized that shorter durations allotted for the go/no-go decision would recruit cerebellar areas when exogenous temporal cues varied [12]. We found that a faster stimulus speed, contrasted with medium speed, was associated with a large cluster in the medial cerebellum (vermis), right lateral cerebellar cortex (crus I) (*Zmax* = 4.01) and the left inferior parietal lobe (*Zmax* = 4.19) (Fig. 3a, b; Table 1). When contrasting only hit responses during fast and medium stimuli speeds, we observed clusters of activity in the medial and right lateral cerebellum (*Zmax* = 3.81) (Fig. 3c; Table 1). When contrasting only correct rejections during fast and medium stimulus speeds, we observed differential activity in the vermis and bilateral cerebellar cortex crus I and II (*Zmax* = 4.19), in addition to the bilateral inferior parietal lobe (*Zmax* = 3.96), bilateral somatomotor cortex (*Zmax* = 4.07), left frontal operculum (*Zmax* = 4.1), and middle frontal gyrus (*Zmax* = 4.25) (Fig. 3d; Table 1). These results indicate that our task recruits both medial and lateral cerebellar regions that are co-active with a network of cortical areas when temporal conditions change, regardless of whether or not a motor response is required for the correct response.

**Table 1:** *Zmax* results contrasting all fast and medium trials, fast and medium hits, and fast and medium correct rejections

Area	Size	*Zmax*	*x*	*y*	*z*
Figure 3a, b Fast–medium					
Cerebellar vermis, bilateral cerebellar cortex VI, VII, crus I	1047	4.01	0	− 72	− 32
Left temporo-occipital cortex, inferior parietal lobule	639	4.19	− 44	− 76	6
Figure 3c Hits fast–medium					
Cerebellar vermis, right cerebellar cortex I–VII, crus I	344	3.81	6	− 54	− 12
Figure 3d Correct rejection fast–medium					
Vermis and bilateral cerebellar cortex I–IX, crus I, crus II	2170	4.19	6	− 58	− 48
Left putamen, left frontal operculum	967	4.1	− 26	6	4
Left temporo-occipital cortex, inferior parietal lobule	925	4.22	− 40	− 74	2
Bilateral superior parietal gyrus, intraparietal sulcus	837	4.07	12	− 62	50
Right temporo-occipital cortex, inferior parietal lobule	537	3.96	54	− 58	4
Middle frontal gyrus	364	4.25	− 34	36	42

### Experts Show Cognitive Efficiency When Fixation Duration Is Short

**Fig. 4:**
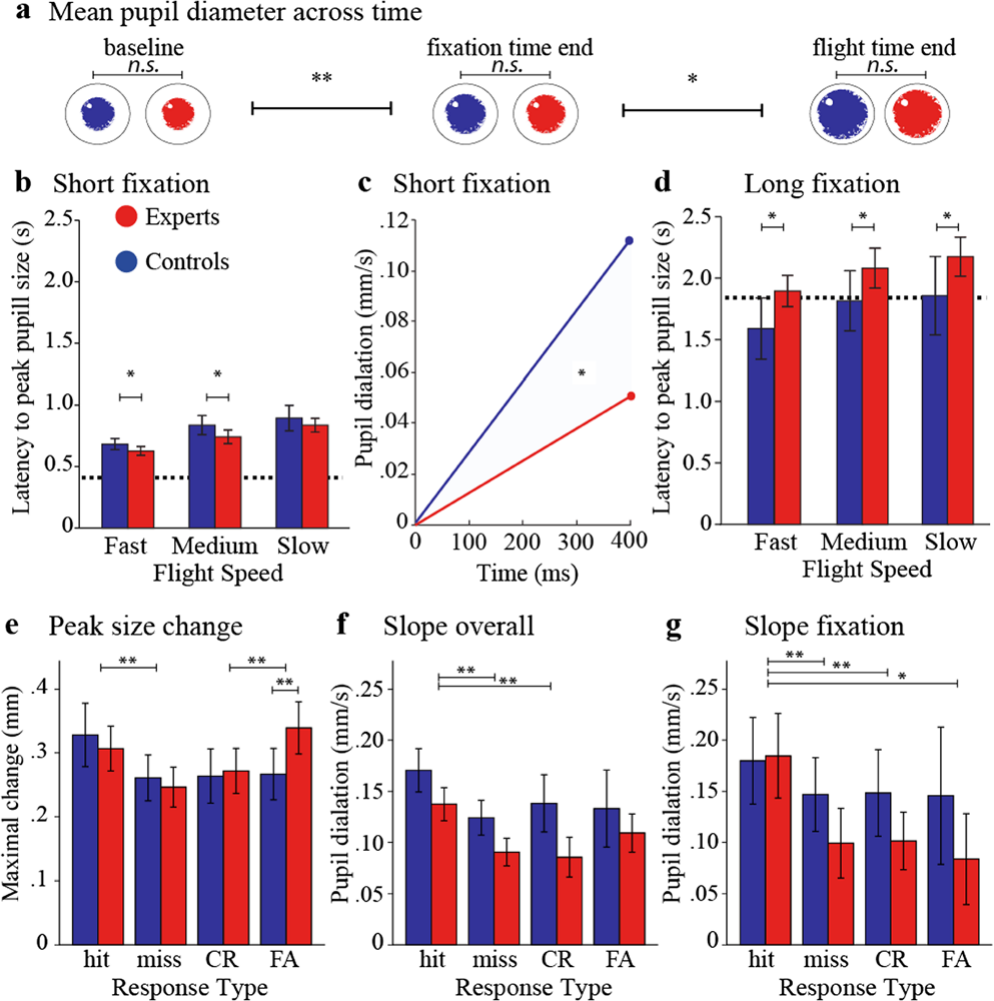
Pupillary responses. Dashed line indicates stimulus onset. **a** Subject pupil diameter increased progressively but did not differ between groups at either baseline, end of fixation time, or end of flight time. **b** During trials with the short fixation, experts reached peak pupil dilation earlier than controls on the fast and medium but not slow flight speeds. **c** Pupil dilation slope during ball flight on trials with the short fixation and fast speed: consistent with peak latencies, expert pupil diameter enlarged less abruptly during flight time. **d** During trials with the long fixation, experts reached peak pupil dilation later than controls at all flight speeds. **e–g** Hit—correct go trials/all go trials; miss—incorrect go trials/all go trials; CR**—**correct rejections: correct no-go trials/all no-go trials; FA—false alarms: incorrect no-go trials/all no-go trials. **e** Experts showed larger dilations during false alarms relative to controls. **f** The slope of pupil dilation across the whole trial (fixation plus flight time) was different when comparing hits to misses and hits to correct rejections. **g** When considering the fixation intervals only, the slope of pupil dilation depended on the response subjects chose at the end of the trial. (*N* = 9/group)

We found task-evoked pupillary responses (TEPRs) to be a reliable measure with consistent baselines between groups at all experimental periods. We observed an increase in pupil size from the beginning to the end of the fixation period [*F* (2, 18) = 16.710, *P* = .003] and between the end of the fixation period and the end of the flight time[*F* (2, 18) = 10.694, *P* = .01], with no significant group differences (Fig. 4a)*.*

When fixation time was short, pupil diameter of experts reached its peak earlier than that of controls at fast [MD = 55, *t* (16) = 2.183, *P* = .044] and medium [MD = 95, *t* (16) = 2.328, *P* = .033], but not slow [MD = 58, *t* (16) = 1.147, *P* = .268] flight speeds (Fig. 4b). Experts also showed a shallower slope of dilation during ball flight at the fastest flight speed [MD = 0.153, *t* (16) = 2.301, *P* = .035] (Fig. 4c), but not at the medium or slow flight speed [MD = 0.087, *t* (16) = 1.251, *P* = .229; MD = 0.028, *t* (16) = 0.311, *P* = .760], indicating that their pupil diameter increased less abruptly once the fast stimulus had appeared. These data show how expert pupil dilation, although comparable to that of controls in size, occurs faster prior to stimulus onset suggesting a more prompt mental engagement that coincides with the preparatory signal.

Since experts are faster in the mobilization of cognitive resources, conditions requiring prolonged or sustained cognitive engagement might exert an additional cognitive load upon them. Indeed, we found that during trials with long fixation durations experts showed a longer latency to peak at all flight speeds (fast [MD = − 306, *t* (16) = − 2.522, *P* = .012, one-tailed], medium [MD = − 265, *t* (16) = − 2.086, *P* = .026, one-tailed] and slow [MD = − 317, *t* (16) = − 2.047, *P* = .028, one-tailed], Fig. 4d). Moreover, the overall dilation slope of experts was steeper at the slow flight speed [MD = − 0.045, *t* (16) = − 1.985, *P* = .032], but not at the medium or fast flight speed [MD = − 0.006, *t* (16)= − 0.241, *P* = .813; MD = 0.004, *t* (16) = 0.100, *P* = .921]. Finally, there were no group differences in peak pupil dilation or slope for intermediate fixation times.

### Experts Show Greater Recruitment of Cognitive Resources During False Alarms

To investigate the relation between pupil size and response types, we arranged and analyzed trials by hits, misses, correct rejections, and false alarms. We found a main effect of response type [*F* (3, 45) = 14.534, *P* < .001] as well as an interaction between response type and group [*F* (3, 45) = 4.707, *P* = .006], suggesting that the extent to which pupil size changes is a function of the response depending on the group. Contrasts revealed that greater pupil dilation occurred with hits compared to misses [*F* (1, 15) = 24.795, *P* < .001] and with false alarms compared to correct rejections [*F* (1, 15) = 15.722, *P* = .001], but not misses compared to correct rejections [*F* (1, 15) = 3.670, *P* = .075] (Fig. 4e). These response type differences may be due to movement preparation and action associated with hits and false alarms. Interestingly, experts showed larger peak dilation for false alarms compared to hits relative to controls [*F* (1, 15) = 0.027, *P* = .007], suggesting the highest level of arousal during incorrect no-go trials.

We found a main effect of response type on the overall dilation slope [*F* (2, 30) = 5195, *P* = .011], meaning that the steepness of pupil dilation across the entire trial varied depending on the choice the subject was about to make. However, there was no interaction between response type and group in this respect. Contrasts revealed that pupil change across the whole trial was significantly different when comparing hits to misses [*F* (1, 15) = 10.033, *P* = .006] and hits to correct rejections [*F* (1, 15) = 66.797, *P* < .001], while differences between dilation slopes associated with false alarms and those associated to the other response types were not significant (Fig. 4f).

Additionally, the main effect of response type on the fixation slope was also significant [*F* (2, 30) = 3.329, *P* = .028], suggesting that the steepness of pupil dilation during fixation time varied according to the response subjects *would* choose later in the trial. There was no interaction between response type and group. Contrasts revealed that change in pupil dilation within the fixation period was steeper for hits relative to misses [*F* (1, 15) = 11.695, *P* = .004], correct rejections [*F* (1, 15) = 24.295, *P* < .001], and false alarms [*F* (1, 15) = 4.920, *P* = .042] (Fig. 4g). These results could not be confounded by motor preparation or action, because the data were only taken during presentation of the fixation cross, the duration of which was pseudo-randomly distributed.

### Experts Show Precise, Cost-Efficient Eye Movements

**Fig. 5:**
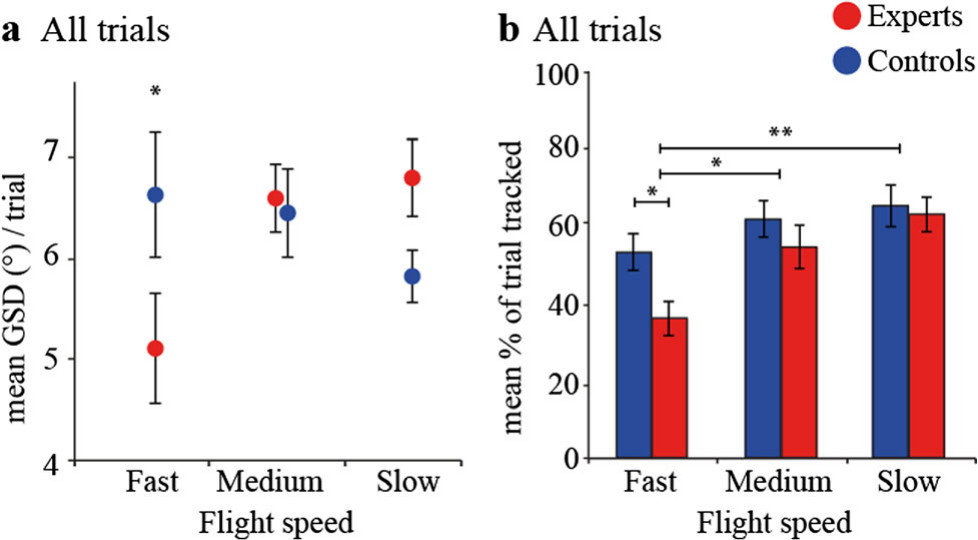
Eye movements. **a** Expert mean gaze-stimulus distance (GSD) was shorter at the fast flight speed and increased as flight speed decreased, while control mean GSD decreased as flight time decreased, showing an interaction between group and flight speed. **b** Tracking measures (see methods) showed a significantly larger percentage of the trial tracked by controls during the fastest flight times. We also found the mean percent of the trial tracked increased as stimulus speed decreased from the fast to medium and the fast to slow flight speed (*N* = 10/group)

Next, we measured eye movements and we found that distance between gaze and stimulus (GSD) during the trials showed an interaction-effect between flight speed and group [*F* (5, 59) = 4.012, *P* = .024; ANOVA] (Fig. 5a). During the fastest flight speed, experts tracked the stimulus for a lower percentage of the trial duration (*F* (1, 54) = 4.733, *P* = .034) (Fig. 5b). These results indicate that during the most challenging trials experts keep their gaze closer to the stimulus initially, but track the stimulus for less of the trial.

To rule out that differences in reaction time contributed to our results, we compared performance of both groups on a simple reaction time task (RT). We did not find significant differences in reaction time between experts (310 ± 1.88 ms) and controls (312 ± 2.29 ms; *t* (18) = 0.264, *P* = .26). All motoric measures including saccade onset, initiation of hand movements, and task reaction time adapted to faster temporal parameters but did not differ between groups.

We also calculated the distance of the subjects’ gaze to the fixation cross at the end of the fixation time (and therefore start of the stimulus movement), so as to determine whether subjects kept their gaze on the fixation cross prior to trial start (fixation error). We found no effect of fixation duration on fixation error [*F* (3, 53) = 0.456, *P* = 0.714] but did find an interaction effect of fixation duration and group on fixation error [*F* (3, 54) = 3.587, *P* = 0.019]. When we compared experts and controls at each fixation duration parameter, we found fixation error was smaller in experts only in trials with the long fixation duration [*t* (9) = 3.874, *P* = 0.024]. These data are in line with the pupillary response results on the long fixation durations, described above.

## Discussion

**Fig. 6:**
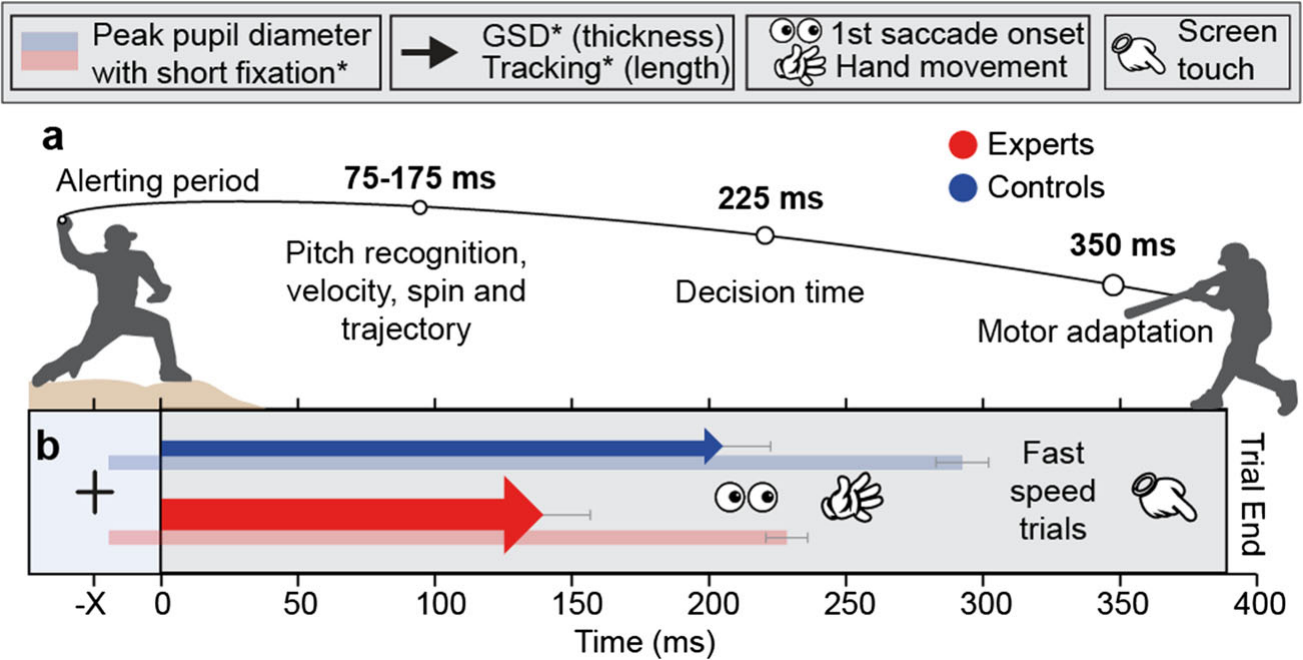
Summary and temporal dynamics. **a** Upper panel: temporal dynamics of a 90-mph professional baseball pitch. **b** Group averages of psychophysical responses at the short preparation and fast stimulus speed trials including significant differences in stimulus tracking (arrow length), gaze to stimulus distance (arrow thickness), and peak pupil dilation (transparent bars). Saccade onset, hand movements, and screen touch reaction times did not differ between groups

We developed a novel trajectory prediction, go/no-go task with the aim of modeling temporal parameters from a hitter’s perspective in baseball so that the response time window reflected a 90-, 80-, or 70-mile/h fastball. The first part of a real pitch necessitates pattern and trajectory prediction followed by a go/no-go decision, while the latter part of the pitch requires precise motor control and timing [6] (Fig. 6a). In our task, subjects were instructed to predict whether a downward-moving stimulus would terminate inside (go trial) or outside (no-go trial) a visible target area on a computer screen. Task difficulty changed by manipulating the duration of temporal cues; three initial fixation intervals, followed by three stimulus speeds pseudo-randomly intermixed. We found medial and lateral cerebellum was activated when contrasting fast trials with medium trials. We also found that experts performed better during the most difficult parameter combinations, i.e., at short fixation duration and fast stimulus speed. Closer gaze-stimulus distance and shorter tracking accompanied superior expert performance measures during ball flight. At short fixation duration, experts exhibited earlier and faster pupillary dilation (Fig. 6b), while at long fixation duration, they showed delayed pupillary responses compared to controls. Finally, pupil dynamics were related to performance, particularly when a motor response was involved (hits and false alarms).

### Cortico-cerebellar Networks

We observed cerebellar network activation during both motor and non-motor responses, suggesting that the cerebellar role in our task cannot be attributed to the motor response alone. Experts showed pronounced performance differences, coinciding with distinct eye movement and pupil dynamics. These findings could reflect tuning of large cortico-cerebellar networks in experts, which in effect allows for quick cognitive preparation to make fast decisions. Recent evidence suggests that baseball players show expertise-level differences in cognitive processing and differences in structural connectivity, supporting functional connectivity and modulatory attention [13].

There is evidence that the basal ganglia (BG) are associated with action selection [14], whereas the cerebellum is more commonly associated with action preparation and execution [15]. When we broke down our data into response types, we found that as flight speed increased, both groups’ hit rates significantly decreased while false alarm rates stayed the same. Therefore in our task, the difference in response windows from 585 to 485 or 385 ms response windows resulted in subjects’ decreased hits and increased misses, indicating increased demands on action execution over action selection. When we analyzed pupil response broken down into response types, we found overall, larger peak pupil dilation in trials where action preparation occurred (hits and false alarms) and that experts had a larger peak dilation during false alarms when compared to controls suggesting a higher level of arousal during incorrect no-go trials. These data indicated to us that action preparation and initiation may influence our group differences in this task to a greater extent than action selection. This evidence also supports our fMRI findings where when contrasting the same response types (hits or false alarms) with differing flight speeds (fast and medium), we found both medial and lateral cerebellar activations. Recent alternative BG hypotheses posit that BG controls a time varying signal that controls speed-accuracy tradeoff and that its activity reflects the commitment to a decision [16]. However, transient increases in deep cerebellar nuclei activity precede and are time-locked to saccades [17] or limb movements in a simple reaction time task [18], both functions required for our task.

Furthermore, cerebellar Purkinje cell simple spike activity has been found to signal visual events and encode target motion and direction in similar visually guided tasks [19, 20], facilitating direct and active integration between perceptual and motor demands [21]. To this extent, there is growing evidence that subcortical circuits may provide a short-cut to drive motor actions prior to visual information reaching awareness [22]. Given that the lateral cerebellum was activated during correct rejections, our data provide evidence that the cerebellum is also involved in the decision process of the initiation of movements, rather than online movement control alone. Although we did not find activation of BG in our fMRI results, we were interested in neural networks involved in the dynamic feedback in the task over the response types themselves. Overall, we conclude that experience in elite sports allows for early activation of arousal systems to precisely tune cortico-cerebellar pathways, resulting in distinct physiological responses required for coping with subsecond decisions.

### Timing

In baseball, a batter must take into account several kinds of information for trajectory interpolation. They are performing an explicit timing task where they must deliberately and exactly time their swing of the bat to coincide with the speed and trajectory of the ball. Since we did not ask our subjects to make an overt estimate of the trial duration, our task did not involve explicit timing. We chose to exclude this real world scenario from the equation, allowing our subjects to decide anywhere within the time of the trial. Still, the feedback response in our task might have contributed to both explicit and implicit learning [23]. Moreover, implicit timing, such as that employed by a batter when observing the angle and movement of the pitchers arm, might be used to predict the forthcoming duration of the stimulus, in a different way than the batter interprets speed based on the perceptual increase in size of the ball approaching.

Although the three fixation durations employed in the current task were not predictable at first, after time it might have been possible to implicitly learn their duration since there were only three. Pupil responses function as reporter indicators for dynamic, intensive aspects of human cognition where the amplitude is proportional to task complexity, allowing one to discriminate individual differences in resource availability and investment [24–26]. Experts performed better than controls when fixation presentation was short and stimulus speed was fast, while they activated cognitive resources faster during these trials. When fixation was long however, they performed similarly but delayed activation of cognitive resources. These results indicate that experts may rely more heavily on endogenous alerting temporal cues than controls.

Optimal feedback control theory attributes a forward model to account for accurate prediction of sensory outcomes from motor commands, such as those for eye movements and hand movements. Such sensory predictions are then integrated in order to estimate the state of the body in the world (state estimation). For a particular estimated state, the system must adjust the gains of sensorimotor feedback loops so movements optimally balance costs and rewards for maximum gain or performance [27]. We found that all subjects adjusted eye, hand, and touch latencies relative to our temporal cues during the trial (flight speed), and these adjustments were strikingly similar across groups. However, experts performed more accurately with earlier cognitive engagement, suggesting experts exhibited optimal preparation skills but similar motor control for this task.

#### Summary and Functional Implications

We have highlighted variable contributions of temporal cues to identify potential visuo-motor and perceptual differences in a population of elite athletes playing a popular sport. Given that experts exhibited superior performance when decision time was short and fixation duration was limited, we purport that the arousal systems of experts may precisely tune the relevant cortico-cerebellar pathways to optimize their psychophysical responses, allowing them to cope with changing task conditions. Indeed, evidence indicates pupil size is associated with the locus coeruleus, superior colliculus, and anterior cingulate cortex and may be crucial for synchronizing arousal states to facilitate behavior [28]. The finding that experts track the ball for less time, but do so more accurately while performing better at the fastest flight speed, may also provide a clue as to how they cope with real-world challenging conditions in their sport. Possibly, experts’ increased exposure to these temporal constraints allows for faster adaptation of eye movements. Cerebellum and the superior colliculus provide directional drive of the eyes, while the cerebellum keeps track of the progress of the saccade toward the target and ends saccades by cutting off drive from the superior colliculus [29]. Purkinje cell simple spike activity has been found to signal visual events and encode target motion in a visually guided reaching task [19], facilitating direct and active integration between perceptual and motor tasks [21]. Intriguingly, accumulating reports argue that the retinocollicular pathway could provide a short-cut to drive motor actions, such as fast orienting eye movements to targets of interest, before visual information reaches awareness [22]. Within our task, this network may be primed in experts in order to make a fast decision accounting for less tracking and at the same time, a better performance on the fastest flight speeds. The retinogeniculate pathway through primary visual cortex (V1) is specialized for feature based motion perception, while the retinocollicular pathway that bypasses V1 is thought to be specialized for detecting motion energy [30].

Taken together, the current study may have multiple and widespread implications toward better neural training methods for elite athletes. Additionally, modeling other sports with control of similar single trial tasks and populations with parametrically diverse cognitive skills may further facilitate a mechanistic understanding to improve rehabilitation for clinical populations.
